# Correlação entre Cardiomegalia pela Radiografia de Tórax e Diâmetro do Ventrículo Esquerdo pela Ecocardiografia em Pacientes com Doença de Chagas

**DOI:** 10.36660/abc.20190673

**Published:** 2021-01-27

**Authors:** Matheus Rassi Fernandes Ramos, Henrique Turin Moreira, Gustavo Jardim Volpe, Minna Romano, Benedito Carlos Maciel, André Schmidt, Anis Rassi, Jose Antônio Marin

**Affiliations:** 1 Universidade de São Paulo Faculdade de Medicina de Ribeirão Preto Ribeirão PretoSP Brasil Universidade de São Paulo Faculdade de Medicina de Ribeirão Preto – Cardiologia, Ribeirão Preto, SP - Brasil; 2 Universidade de São Paulo Faculdade de Medicina de Ribeirão Preto Ribeirão PretoSP Brasil Universidade de São Paulo Faculdade de Medicina de Ribeirão Preto – Medicina Interna, Ribeirão Preto, SP - Brasil; 3 Hospital do Coração Anis Rassi GoiâniaGO Brasil Hospital do Coração Anis Rassi – Cardiologia, Goiânia, GO – Brasil

**Keywords:** Doença de Chagas/fisiopatologia, Cardiomegalia, RX/ Tórax, Cardiomiopatia Chagásica, Bloqueio Cardíaco

## Abstract

**Fundamento:**

Cardiomegalia pela radiografia de tórax (RXT) é preditor independente de morte em indivíduos com cardiomiopatia crônica da doença de Chagas (CCDC). Contudo, a correlação entre o aumento do índice cardiotorácico (ICT) na RXT e do diâmetro telediastólico do ventrículo esquerdo (DDVE) pela ecocardiografia (ECO) nessa população não está bem definida.

**Objetivos:**

Analisar a relação entre cardiomegalia pela RXT e DDVE pela ECO em pacientes com doença de Chagas (DC) e sua aplicabilidade ao escore de Rassi.

**Métodos:**

Estudo retrospectivo incluiu 63 pacientes ambulatoriais com DC avaliados por RXT e ECO. Cardiomegalia na RXT foi definida como ICT > 0,5. DDVE foi avaliado como variável contínua. Curva ROC foi utilizada para avaliar o potencial do DDVE para identificação de cardiomegalia pela RXT, com ponto de corte definido pela maior somatória de sensibilidade e especificidade.

**Resultados:**

Idade mediana = 61 anos [intervalo interquartil: 48-68], 56% mulheres. CCDC foi identificada em 58 pacientes; 5 tinham a forma indeterminada da DC. Cardiomegalia foi detectada em 28 indivíduos. A área sob a curva ROC do DDVE para identificação de cardiomegalia foi de 0,806 (IC 95%: 0,692-0,919). O ponto de corte ótimo para DDVE foi de 60 mm (sensibilidade = 64%, especificidade = 89%). O uso do DDVE pela ECO em substituição ao ICT pela RXT alterou o escore de Rassi em 14 pacientes, e em 10 deles houve redução do risco presumido.

**Conclusão:**

DDVE pela ECO é parâmetro adequado e com alta especificidade para distinguir entre presença e ausência de cardiomegalia na RXT na DC. (Arq Bras Cardiol. 2021; 116(1):68-74)

## Introdução

A doença de Chagas (DC) resulta de infecção pelo protozoário *Trypanosoma cruzi* ( *T. cruzi* ), cujo principal vetor de transmissão ao ser humano são insetos da subfamília *Triatominae,* embora isso possa ocorrer por outros mecanismos, tais como transfusão sanguínea, transplante de medula óssea ou de órgãos sólidos a partir de doadores contaminados, transmissão vertical entre mãe e filho e ingestão de alimentos contaminados.^1^ Estimativas da Organização Mundial da Saúde (OMS) reportam que aproximadamente 7 milhões de indivíduos estão infectados mundialmente, proporcionando elevada morbimortalidade e impacto social.^2^

A cardiomiopatia é a mais séria e frequente forma clínica da DC crônica, que ocorre em 20% a 30% dos indivíduos cronicamente infectados.^3 , 4^ O escore de Rassi é estratificador do risco de morte validado em pacientes com cardiomiopatia crônica da DC (CCDC). Dentre os fatores de risco que compõem esse escore, destaca-se a cardiomegalia na radiografia de tórax (RXT), que se associa a significativo risco de morte total e cardiovascular em pacientes com a CCDC.^5^

Embora o estudo de Rassi Jr et al.^5^ tenha utilizado a ecocardiografia (ECO) para determinar o diâmetro telediastólico do ventrículo esquerdo (DDVE), esse parâmetro não se mostrou marcador independente de mortalidade na CCDC. Entretanto, deve-se ressaltar que, naquele estudo, o DDVE foi avaliado de maneira categórica, com pontos de corte convencionais que podem não ser os mais adequados para pacientes com CCDC, devido ao típico envolvimento miocárdico segmentar nessa doença. De outra parte, o cálculo do índice cardiotorácico (ICT) pela RXT pode englobar, em muitos casos, a dilatação de átrios e ventrículos, que se expressam linearmente na RXT. Embora o estudo radiológico do coração seja bastante disponível, implica radiação, enquanto a ECO tornou-se atualmente um método amplamente utilizado para avaliação cardiovascular. Dessa maneira, haveria indiscutível interesse em, com um único exame, avaliar função sistólica e dimensão do ventrículo esquerdo (VE), a fim de estimar o risco de morte com variáveis correlatas às aplicadas no escore de Rassi original.

O presente estudo teve por objetivo analisar a relação entre cardiomegalia definida pelo ICT na RXT e o DDVE avaliado com ECO em pacientes com DC.

## Métodos

Neste estudo transversal retrospectivo, foram incluídos pacientes de ambos os gêneros, adultos (> 18 anos de idade), com diagnóstico de DC, caracterizados por positividade em dois exames sorológicos para detecção de anticorpos contra o *T. cruzi* , embasados em técnicas distintas, que se encontravam em seguimento ambulatorial no Hospital das Clínicas da Faculdade de Medicina da Universidade de São Paulo em Ribeirão Preto (HCFMRP-USP), hospital terciário e centro de referência em pesquisa de DC.

A coleta de dados foi realizada por meio de revisão sistematizada de prontuários clínicos, a partir de um grupo de 158 pacientes com DC que participaram de estudo clínico prévio, cujos critérios de inclusão e exclusão foram anteriormente reportados em detalhes por outra publicação.^6^ Foram incluídos neste estudo os pacientes que tivessem RXT e avaliação completa dos preditores independentes do escore de Rassi, mediante os exames essenciais de eletrocardiograma de 12 derivações, em repouso, ECO, RXT e exame de monitoramento de 24 horas do ritmo cardíaco, além de anamnese focada na gradação de dispneia conforme a New York Heart Association (NYHA). Para inclusão no estudo, o limite máximo de tempo entre a realização da RXT, considerada como método comparativo de referência, e a ECO foi de 1 ano, desde que o *status* clínico do paciente tivesse se mantido estável nesse período. Foram excluídos da análise os pacientes que apresentaram mudança no quadro clínico no decorrer do período entre a realização desses dois exames.

### Radiografia de Tórax

A cardiomegalia na RXT foi sempre avaliada na incidência posteroanterior e definida como a presença de ICT > 0,5. Foi envidado esforço adicional para eventual caracterização de aumento ventricular direito, por comparação com a telerradiografia em perfil, nos casos em que essa incidência radiológica estava disponível.

### Ecocardiografia

Foram utilizados os dados de ECO transtorácica em repouso, realizada na data mais próxima à da RXT, desde que respeitado o período máximo de 1 ano. O DDVE foi mensurado pelo método bidimensional, seguindo diretrizes ecocardiográficas recentes.^7^ Outros parâmetros ecocardiográficos também relacionados ao remodelamento do VE foram analisados, tais como o índice de massa e a fração de ejeção (FE) do VE e o volume indexado do átrio esquerdo.

### Análise Estatística

Variáveis contínuas foram apresentadas como média e desvio padrão quando distribuídas normalmente, e representadas como mediana e intervalo interquartil quando apresentavam distribuição não normal. Análise de curva ROC ( *Receiver Operating Characteristic* ) foi realizada para verificar a capacidade de o DDVE, avaliado pela ECO, distinguir entre presença e ausência de cardiomegalia pela RXT. Por fim, foi avaliado o impacto na reclassificação dos pacientes com a forma cardíaca da DC pelo escore de Rassi, utilizando-se a ECO para a definição de cardiomegalia, em substituição ao tradicional ICT. Os indivíduos com a forma indeterminada da DC não foram classificados pelo escore de Rassi neste estudo, pois pacientes com essa forma da doença não foram contemplados na investigação original desse escore

### Ética

O presente estudo foi aprovado pelo Comitê de Ética em Pesquisa do HCRP-FMRP-USP (CAAE: 06415319.2.0000.5440; parecer 3.130.390) e conduzido sob os princípios éticos da declaração de Helsinque e em conformidade com a resolução do Conselho Nacional de Saúde n^o^466/2012.

## Resultados

### Descrição da Amostra Populacional Estudada

Este estudo transversal retrospectivo incluiu 63 pacientes com DC que preencheram os critérios de inclusão, correspondendo a 40% do total de 158 pacientes avaliados. As características demográficas e clínicas dos participantes estão descritas na Tabela 1 . Os participantes apresentaram idade mediana de 61 anos (intervalo interquartil [IIQ]:48-68), na maioria mulheres (56%). Apenas 5 (8%) pacientes apresentavam a forma indeterminada da DC. A maioria dos participantes (68%) estava em classe funcional I pela NYHA, enquanto classes funcionais II e III foram encontradas em 21% e 11% dos indivíduos, respectivamente. O escore de Rassi calculado nos 58 pacientes com a CCDC foi de 9 ± 5 pontos.

**Tabela 1:** – Características dos pacientes incluídos no estudo

Demográficos e antropométricos	
Idade (anos)	61 [48-68]
Gênero feminino	35 (56%)
IMC (kg/m^2^)	26,6 ± 4,7
**Clínicos**	
Classe funcional	
NYHA I	37 (59%)
NYHA II	17 (27%)
NYHA III	9 (14%)
Edema de membros inferiores	12 (19%)
Turgência jugular	3 (5%)
IECA ou BRA	48 (76%)
Betabloqueador	34 (54%)
Espironolactona	16 (25%)
Diuréticos	29 (46%)
Amiodarona	14 (22%)
**Ecocardiográficos**	
Diâmetro diastólico final do VE (mm)	54 [47-61]
Volume indexado do átrio esquerdo (mL/m^2^)	42 [26-59]
Índice de massa ventricular (g/m^2^)	123 [92-156]
Fração de ejeção do ventrículo esquerdo (%)	51 [34-63]

*Variáveis contínuas paramétricas e não paramétricas estão descritas como média ± desvio padrão e como mediana [intervalo interquartil], respectivamente. Variáveis categóricas estão representadas como contagens absolutas e porcentagens. IMC: índice de massa corporal; NYHA: New York Heart Association; IECA: inibidor da enzima de conversão da angiotensina; BRA: bloqueador do receptor da angiotensina II; VE: ventrículo esquerdo.*

### Comparação entre Radiografia de Tórax e Ecocardiograma

**Figura 1:**
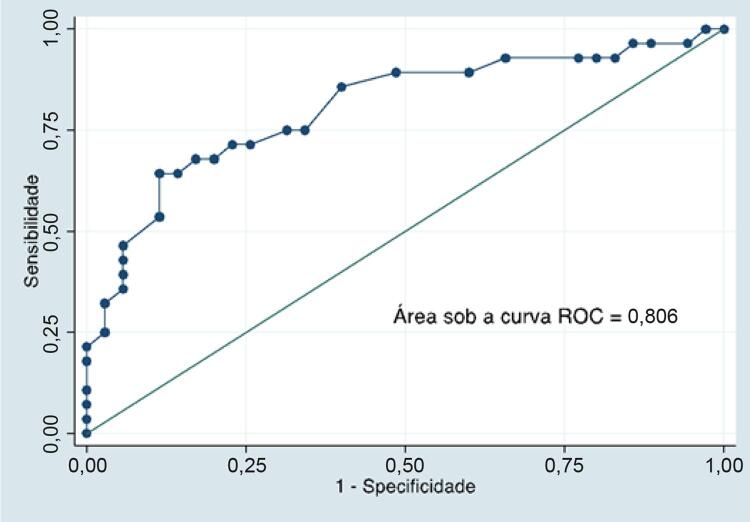
– Área sob a curva ROC do diâmetro telediastólico do ventrículo esquerdo para a detecção de cardiomegalia em radiografia de tórax.

Cardiomegalia foi detectada pela RXT em 28 (44%) indivíduos. O tempo médio entre a realização da RXT e da ECO foi de 5 ± 174 dias. O DDVE dos indivíduos no grupo com cardiomegalia (61 IIQ [53-70]) pela RXT mostrou-se maior do que no grupo sem cardiomegalia (49 IIQ [46-55]), p < 0,001. A área sob a curva ROC do DDVE para identificação de cardiomegalia detectada pela RXT foi de 0,806 (intervalo de confiança de 95%: 0,692-0,919) ( Figura 1 ). Na amostra estudada, o DDVE de 60 mm apresentou-se como o ponto de maior acurácia, com sensibilidade de 64% e especificidade de 89%.

Os indivíduos com resultados discordantes para cardiomegalia definida pelos dois métodos de imagem não mostraram diferenças estatisticamente significativas quanto a idade, gênero, índice de massa ventricular esquerda e volume indexado do átrio esquerdo em comparação aos indivíduos com resultados concordantes.

### Reclassificação do Escore de Rassi

**Figura 2:**
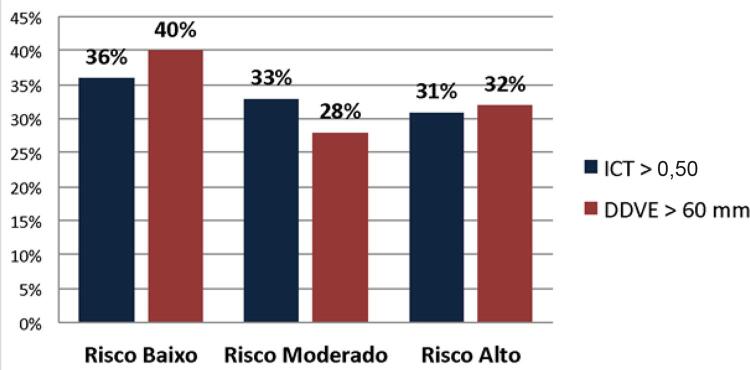
– Classificação de risco de acordo com o escore de Rassi utilizando a radiografia de tórax e a ecocardiografia para definir cardiomegalia nos indivíduos com a forma cardíaca da doença de Chagas. DDVE: diâmetro diastólico final do ventrículo esquerdo pela ecocardiografia; ICT: índice cardiotorácico pela radiografia de tórax.

**Figura 3:**
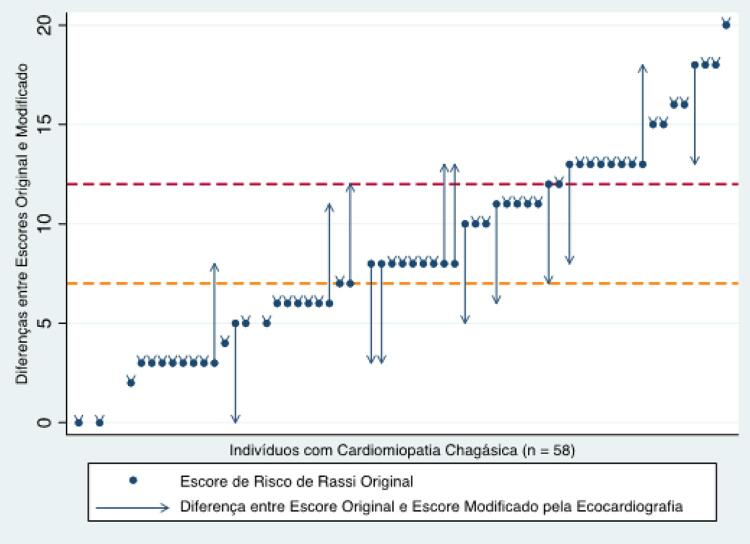
– Classificação de risco individual de acordo com o escore de Rassi utilizando a radiografia de tórax e a ecocardiografia para definir cardiomegalia nos indivíduos com a forma cardíaca da doença de Chagas. Linhas tracejadas amarela e vermelha correspondem, respectivamente, a escore de Rassi = 7 (risco moderado) e a escore de Rassi = 12 (risco alto). DDVE: diâmetro diastólico final do ventrículo esquerdo pela ecocardiografia; ICT: índice cardiotorácico pela radiografia de tórax.

Na presente amostra populacional, a proporção de indivíduos considerados de risco baixo, moderado e alto de morte pelo escore de Rassi, utilizando a RXT para identificação de cardiomegalia, foi de 36% (n = 21), 33% (n = 19) e 31% (n = 18), respectivamente. Ao utilizar-se a ECO para identificação de cardiomegalia, com o ponto de corte de maior acurácia, a proporção de pacientes com risco baixo, moderado e alto foi de 40% (n = 23), 28% (n = 16) e 32% (n = 19), respectivamente ( Figura 2 ). Em 44 (76%) participantes, o escore de Rassi com a utilização da ECO mostrou o mesmo valor numérico quando comparado com o escore estimado por utilização da RXT. Dentre os 14 participantes em que houve mudança numérica do escore, 8 demonstraram redução do mesmo, enquanto 6 demonstraram aumento. Quanto às categorias de risco (baixo, moderado, elevado), houve alteração com a utilização da ECO em 11 pacientes (19%), 5 deles com aumento e 6 deles com redução do risco atribuído pelo escore assim constituído ( Figura 3 ).

## Discussão

Este estudo demonstrou existir relação bastante nítida e significativa entre a detecção de cardiomegalia pela RXT e a verificação de dilatação ventricular esquerda, pela ECO, em amostra não selecionada de pacientes ambulatoriais com diagnóstico de CCDC. Esses resultados abrem a perspectiva para que o DDVE avaliado na ECO transtorácica em repouso possa substituir a aferição do ICT na RXT, no sentido de se determinar o risco de morte em pacientes com a CCDC com o escore de Rassi, assim eventualmente modificado. Isso permitiria dispensar o exame radiológico que requer irradiação do paciente, embora de pequena monta, e determinar com apenas um exame (também não invasivo, mas que, além disso, ainda dispensa irradiação) tanto a função sistólica ventricular esquerda quanto a dimensão dessa câmara. Essa perspectiva torna-se atraente, uma vez que o DDVE, pela ECO, constituiu parâmetro adequado, com alta especificidade, para distinguir entre presença e ausência de cardiomegalia em pacientes com DC crônica.

Os resultados da presente investigação corroboram os de pesquisa anterior relatada em 1983 por Pereira-Barreto et al.,^8^ com amostra bem menor, composta por 22 pacientes, que também focalizou resultados da RXT e da ECO e concluiu por boa correlação entre os valores de ICT e de função ventricular esquerda.^8^ Em contraposição, outro estudo, por Perez et al. em 2003, relatou sobre análise comparativa entre os resultados da RXT em incidência posteroanterior e os da ECO em repouso.^9^ Diversamente dos resultados de nosso estudo, aqueles pesquisadores descreveram baixa correlação de valores de ICT pela RXT, tanto com o DDVE quanto com a FE do VE. Além disso, o ICT naquela pesquisa não demonstrou ter elevados índices de sensibilidade ou valor preditivo positivo para detectar disfunção ventricular esquerda. Dessa maneira, concluiu-se que o ICT anormal (> 0,5) apesar de elevada especificidade para detectar a disfunção sistólica do VE, não seria exame útil na avaliação dessa anormalidade na DC, em vista de sua baixa sensibilidade para detectar tanto a dilatação ventricular avaliada pela ECO transtorácica em repouso quanto a disfunção sistólica da câmara, medida pela FE do VE com o mesmo método.

Não são claros os possíveis motivos para a discrepância entre os resultados do presente estudo e aqueles relatados por Perez et al.^9^ Ambos os estudos são embasados em amostras populacionais de pacientes com CCDC que eram ambulatoriais, orbitando em ambientes de hospitais universitários, mas há também alguns aspectos diferenciais entre as amostras estudadas. Assim, não houve praticamente critérios de exclusão em nosso estudo, salvo os de conveniência logística, decorrentes da disponibilidade dos dois exames comparativos em intervalo de tempo prefixado de 1 ano. Em contraste, os critérios de exclusão na pesquisa de Perez et al.^9^ foram bastante amplos e abrangentes, embora aplicados a pacientes sucessivos, e possivelmente resultaram em amostra relativamente selecionada e menos representativa da população de pacientes com DC. Assim, naquele estudo, apenas 28% e 29% dos pacientes apresentavam, respectivamente, aumento do ICT e disfunção ventricular esquerda. Em contraste, em nosso estudo, 44% dos pacientes exibiam aumento do ICT. Ademais, no estudo de Perez et al.,^9^ o valor médio de FE do VE foi de 61%; enquanto, na presente investigação, esse parâmetro foi em média bem menor, de 51%. Em conjunto, tais aspectos configuram maior gravidade da CCDC em nossa amostra populacional estudada, incluindo o fato de apenas 8% de indivíduos estarem com a forma indeterminada da DC.

Ainda comparando os resultados da presente pesquisa com a relatada por Perez et al.,^9^ é também plausível inferir que a discrepância de conclusões quanto ao valor da RXT seja mais aparente do que real. De fato, quando aqueles autores dividiram sua amostra em dois grupos, com ICT normal e anormal (> 0,5), seus resultados evidenciaram que o aumento de ICT associou-se significativamente (p < 0,05) com queda da FE do VE, e com aumento da dimensão diastólica ventricular, bem como com a disfunção segmentar do VE, como avaliada por índice de mobilidade parietal ventricular esquerda.

É relevante ressaltar que os resultados da presente pesquisa indicam que a ausência da cardiomegalia ao RXT não descarta comprometimento cardíaco quando pacientes são avaliados por métodos capazes de prover mais detalhes anatômicos e funcionais como a ECO transtorácica em repouso. Portanto, embora específica, a RXT apresenta baixa sensibilidade para detectar acometimento cardíaco em pacientes com DC, e pode ser questionado seu uso como exame de rastreio e como critério para diagnóstico de forma indeterminada da DC. Nesse contexto, deve-se observar que, embora a forma indeterminada da DC continue a ser definida em consensos recentes com base no conceito de uma RXT evidenciando ICT normal,^3 , 10 , 11^ uma proposta de substituição desse critério por ecocardiograma em repouso dentro da normalidade já foi publicada em 2002.^12^

O escore de Rassi é universalmente entendido nos dias atuais como a ferramenta mais valiosa no estabelecimento do prognóstico vital de pacientes com a CCDC. Trata-se de escore robusto, desenvolvido por análise multivariada de vários fatores simples de risco de morte na CCDC e validado externamente em outras coortes independentes. Entretanto, deve-se observar que o estudo em questão abordou variáveis de maneira dicotomizada e não contínua, o que abre margem para complementações. Dentre as variáveis abordadas de maneira não contínua está o DDVE, mensurado padronizadamente com a ECO. Entretanto, o DDVE, quando avaliado em categorias dicotomizadas de aumento *versus* não aumento, pode não ser apropriado à avaliação de pacientes com a CCDC devido ao típico envolvimento miocárdico segmentar nessa doença.^1^

Os resultados do presente estudo estão alinhados com a ênfase na ECO como instrumento fundamental para o seguimento clínico do paciente com DC crônica, mormente quando já se configura o aparecimento da CCDC.^10^ Além de possibilitar a confirmação (ou não) de cardiomegalia por RXT duvidosas, a ECO torna-se previsivelmente essencial para análise de uma cardiomiopatia em que alterações regionais da contratilidade ventricular são distúrbios precoces e proeminentes na história natural da doença de base.^13^ Ademais, a detecção dessas anormalidades regionais, em fases precoces da evolução da CCDC, passa a ter implicação prognóstica e terapêutica eventual, face aos resultados recentes de pesquisas evidenciando que um escore de mobilidade segmentar mesmo minimamente alterado – e ainda que a função ventricular sistólica global fosse preservada – é determinante para desfechos graves, incluindo mortalidade.^13^

No estudo seminal de Rassi et al.,^5^ coorte incluída para análise multivariada e elaboração do escore foi constituída de 424 indivíduos, com a maioria deles de baixo risco (61%), enquanto aqueles com risco moderado e alto representaram 19% e 20% da amostra, respectivamente. Já no presente estudo, obteve-se distribuição mais equilibrada entre os grupos de risco, com a proporção de indivíduos de risco baixo, intermediário e alto igual a 36%, 33% e 31%, respectivamente. De forma análoga, quando se substituiu o ICT pelo DDVE para composição do escore, registrou-se pequeno aumento da proporção de baixo risco (40%) às custas de discreta redução na de risco intermediário (28%). Dessa forma, torna-se imperativo conduzir estudos futuros (de seguimento clínico) para eventual validação de um escore de Rassi modificado pela substituição do ICT como parâmetro radiológico de dilatação cardíaca pelo mais específico parâmetro ecocardiográfico de dilatação do VE, para se determinar. o impacto prognóstico dessa modificação.

### Limitações

O presente estudo tem algumas limitações. Os exames utilizados de maneira comparativa (RXT e ECO) não foram realizados no mesmo dia. Os outros exames e avaliações pertencentes ao escore de Rassi também diferem em data. De toda maneira, objetivou-se diminuir ao máximo fatores de confusão ou associados a alterações clínicas, de forma a destacar-se que, no período entre os exames, não houve alteração no quadro clínico dos pacientes ou medicamentosa nos tratamentos dos mesmos. Não houve mensuração das dimensões do átrio direito pela ECO, limitando assim a avaliação da dilatação dessa câmara cardíaca como fator de discordância entre a ECO e a RXT para definição de cardiomegalia. Os pacientes não foram avaliados de maneira longitudinal para análise prognóstica do DDVE pela ECO, mas estão em seguimento ambulatorial e, em ocasião propícia futura, serão reavaliados quanto a isso.

## Conclusões

O DDVE pela ECO é parâmetro adequado para distinguir entre presença e ausência de cardiomegalia na RXT, com alta especificidade em pacientes com DC crônica. A substituição do ICT radiológico pela DDVE ecocardiográfica na composição do escore de Rassi revelou-se possível e não alterou substancialmente os valores do escore assim obtidos. Dessa maneira, abre-se relevante perspectiva de evitar um teste que implique radiação e, com um único exame – ECO – obter mensuração de dois constituintes do escore de Rassi (dimensão cardíaca e função sistólica ventricular). O potencial papel prognóstico do escore assim modificado passa a ser alvo preferencial de estudos futuros.
