# Supplementary material for: Correlação entre Cardiomegalia pela Radiografia de Tórax e Diâmetro do Ventrículo Esquerdo pela Ecocardiografia em Pacientes com Doença de Chagas
Source: Arq Bras Cardiol. 2021 Jan 27;116(1):68–74. [Article in Portuguese] doi: 10.36660/abc.20190673 (PMC8159503; doi:10.36660/abc.20190673)
Supplement: Translation

# Translation: Correlation between Cardiomegaly on Chest X-Ray and Left Ventricular Diameter on Echocardiography in Patients with Chagas Disease

**Authors:** Matheus Rassi Fernandes Ramos, Henrique Turin Moreira, Gustavo Jardim Volpe, Minna Romano, Benedito Carlos Maciel, André Schmidt, Anis Rassi, Jose Antônio Marin

**Affiliations:** 1 Brazil Universidade de São Paulo Faculdade de Medicina de Ribeirão Preto – Cardiologia, Ribeirão Preto, SP - Brazil; 2 Brazil Universidade de São Paulo Faculdade de Medicina de Ribeirão Preto – Medicina Interna, Ribeirão Preto, SP - Brazil; 3 Brazil Hospital do Coração Anis Rassi – Cardiologia, Goiânia, GO – Brazil

**Keywords:** Chagas Disease/physiopathology, Cardiomegaly, X-Rays, Chagasic, Cardiomyopathy, Heart Block

## Abstract

**Background:**

Cardiomegaly on chest X-ray is an independent predictor of death in individuals with chronic Chagas cardiomyopathy (CCC). However, the correlation between increased cardiothoracic ratio (CTR) on chest X-ray and left ventricular end-diastolic diameter (LVEDD) on echocardiography is not well established in this population.

**Objectives:**

To assess the relationship between chest X-ray and LVEDD on echocardiography in patients with Chagas disease and its applicability to the Rassi score.

**Methods:**

Retrospective study on 63 Chagas disease outpatients who underwent chest X-ray and echocardiography. Cardiomegaly on chest X-ray was defined as a CTR>0.5. LVEDD was analyzed as a continuous variable. ROC curve was used to evaluate the ability of LVEDD in detecting cardiomegaly by chest X-ray, with a cut-off point defined by the highest sum of sensitivity and specificity.

**Results:**

Median age 61 years [interquartile range 48-68], 56% were women. CCC was detected in 58 patients, five patients had the indeterminate form of Chagas disease. Cardiomegaly was detected in 28 patients. The area under the ROC curve for LVEDD was 0.806 (95%CI: 0.692-0.919). The optimal cut-off for LVEDD was 60 mm (sensitivity = 64%, specificity = 89%). The use of LVEDD on echocardiography as a surrogate for CTR on chest X-ray changed the Rassi score values of 14 patients, with a reduction in the presumed risk in 10 of them.

**Conclusion:**

LVEDD on echocardiography is an appropriate, highly specific parameter to distinguish between the presence and absence of cardiomegaly on chest X-ray in Chagas disease. (Arq Bras Cardiol. 2021; 116(1):68-74)

## Introduction

Chagas disease (CD) is caused by infection with the protozoan parasite *Trypanosoma cruzi* ( *T.cruzi* ), which is mainly transmitted to humans by insects in the subfamily Triatominae. Other modes of transmission include blood transfusion, bone marrow or solid organ transplantation from infected donors, vertical transmission from mother to fetus and oral ingestion of contaminated food.^1^The World Health Organization estimates that CD affects approximately 7 million individuals in the world, causing high morbidity and mortality, and significant social impact.^2^

Chagas cardiomyopathy is the most common and serious clinical form of CD, affecting 20-30% of chronically infected individuals.^3 , 4^The Rassi score is a validated score for mortality risk stratification of patients with chronic Chagas cardiomyopathy (CCC). Among the risk factors assessed by the score, cardiomegaly on chest X-ray stands out for its strong association with overall and cardiovascular mortality risk in patients with CCC.^5^

In the study by Rassi Jr. et al.,^5^although echocardiography was used to assess left ventricular end-diastolic diameter (LVEDD), this parameter was not shown to be an independent marker of mortality in CCC. However, in their study, LVEDD was analyzed in a categorical manner, using conventional cut-off points, which may not be the most appropriate for CCC patients, due to the segmental myocardial dysfunction, characteristic of this condition. Besides, calculation of the cardiothoracic ratio (CTR) by chest X-ray, in many cases, may encompass both atrial and ventricular dilatation, that are expressed linearly in this method. Despite widely available, the radiological study of the heart involves radiation, and echocardiography has become the most used method for cardiovascular evaluation. Therefore, there is a genuine interest in assessing left ventricular size and systolic function to estimate the risk of death using a single imaging test and the variables used in the original Rassi score.

The present study aimed to evaluate the relationship between cardiomegaly defined by the CTR on chest X-ray and the LVEDD determined by echocardiography in patients with CD.

## Methods

In this retrospective cross-sectional study, we studied patients of both sexes, adults (>18 years old), with diagnosis of CD, attending the outpatient clinic of the General Hospital of the University of Sao Paulo Medical School in Ribeirao Preto (HCRP-FMRP-USO), a tertiary referral hospital for CD. The diagnosis of CD was confirmed by two positive serological tests for detection of antibodies against *T. cruzi* , using different techniques.

Data were obtained by a systematic review of medical records of 158 patients who had participated in a previous clinical study,^6^which describes in detail the inclusion and exclusion criteria applied. Patients with CTR and complete evaluation of the independent predictors of the Rassi score by resting 12-lead electrocardiogram, echocardiography, CTR and 24-hour heart rhythm monitoring, and of the degree of dyspnea according to the New York Heart Association (NYHA) criteria were included. The maximum interval between the CTR (considered the reference method for comparison) and the echocardiography was of one year, and patients were clinically stable in this period. Patients who had changes in the clinical status in this period between the two tests were excluded from the study.

### Chest x-ray

Cardiomegaly on chest X-ray was always evaluated in an anteroposterior view and defined as a CTR > 0.5. Eventual enlargement of the right ventricle was also evaluated by comparison with profile teleradiography, when this technique was available.

### Echocardiography

Data of resting transthoracic echocardiography (the last before the chest X-ray) were used for analysis, considering the maximum interval of one year between the tests. The LVEDD was measured by the two-dimensional test, following recent echocardiography guidelines.^7^Other echocardiographic parameters related to left ventricular remodeling were assessed, including the left ventricular mass index (LVMI), left ventricular ejection fraction (LVEF), and the left atrial volume index (LAVI).

## Statistical Analysis

Continuous variables with normal distribution were expressed as mean and standard deviation, and those without normal distribution were expressed as median and interquartile range (IQR). Analysis of the ROC (Receiver Operating Characteristic) was conducted to verify the ability of LVEDD, determined by echocardiography, to differ between presence and absence of cardiomegaly by the CTR. Finally, the impact of using cardiomegaly assessed by echocardiography, rather than the traditional CTR, on reclassification of patients with the cardiac form of CD by the Rassi score was evaluated. Individuals with the indeterminate form of CD were not classified by the Rassi score in the study, since patients with this form of the disease were not included in the original investigation of this instrument.

### Ethics

The present study was approved by the research ethics committee (CAAE number 06415319.2.0000.5440; approval number 3.130.390) and conducted according to the Helsinki declaration and the Brazilian National Health Council resolution number 466/2012.

## Results

### Description of the Study Population Sample

Of the 158 patients with CD evaluated, 63 (40%) patients met the inclusion criteria and were included in this retrospective cross-sectional study. Demographic and clinical characteristics of participants are described in Table 1 . Median age of participants was 61 (IQR 48-68) years, and many were women (56%). Only five (8%) patients had the indeterminate form of CD. Most patients (68%) had NYHA functional class I, followed by NYHA class II (21%) and III (115). The Rassi score of the 58 patients with CCC was 9 ± 5 points.

**Table 1:** – Characteristics of patients included in the study (n=63)

Demographic and anthropometric data	
Age (years)	61 [48-68]
Female sex	35 (56%)
Body mass index (Kg/m^2^)	26.6 ± 4.7
**Clinical data**	
**Functional class**	
NYHA I	37 (59%)
NYHA II	17 (27%)
NYHA III	9 (14%)
Edema of lower limbs	12 (19%)
Swollen jugular vein	3 (5%)
ACEI or ARB	48 (76%)
Betablocker	34 (54%)
Spironolactone	16 (25%)
Diuretics	29 (46%)
Amiodarone	14 (22%)
**Echocardiographic data**	
Left ventricular end-diastolic diameter (mm)	54 [47-61]
Left atrial volume index (mL/m^2^)	42 [26-59]
Left ventricular mass index (g/m^2^)	123 [92-156]
Left ventricular ejection fraction (%)	51 [34-63]

*Parametric and non-parametric continuous variables described as mean ± standard deviation and median [interquartile range], respectively. Categorical variables presented as absolute numbers and percentages NYHA: New York Heart Association; ACEI: angiotensin-converting enzyme inhibitor; ARB: angiotensin II receptor blocker*

### Comparison between Chest Radiography and Echocardiogram

Cardiomegaly, assessed using the CTR, was detected in 28 (44%) patient. The mean interval between chest X-ray and echocardiography was 5 ± 174 days. LVEDD on chest X-ray was larger in the group of patients with cardiomegaly (61 IQR [53-70]) than in patients without cardiomegaly (49 IQR [46-55]), p < 0.001. The area under the ROC curve for LVEDD for detection of cardiomegaly on chest X-ray was 0.806 (95% confidence interval 0.692 – 0.919) ( Figure 1 ). A LVEDD of 60 mm was defined as the cut-off with the highest accuracy, with sensitivity of 64% and specificity of 89%.

**Figure 1:** – Area under the ROC curve of the left ventricular end-diastolic diameter for detection of cardiomegaly on chest radiography.

Patients with discordant test results regarding the detection of cardiomegaly did not show statistically significant differences in age, sex, LVMI, and LAVI compared with those patients with concordant test results.

### Reclassification of the Rassi Score

In our study, the proportion of individuals at low, moderate and high risk by the Rassi score, using the CTR for detection of cardiomegaly was 36% (n = 21), 33% (n = 19) and 31% (n = 18), respectively. These proportions were 40% (n = 23), 28% (n = 16) and 32% (n = 19), respectively when cardiomegaly was detected by echocardiography using the most accurate cut-off point ( Figure 2 ). In 44 (76%) patients, the Rassi score with echocardiography was the same as that estimated by the CTR. Among the 14 patients who showed a numerical change in the Rassi score, eight showed a reduction in the score and six showed an increase. Considering the risk categories (low, moderate, high), there was a change in category in 11 patients (19%); six showed a reduction and five showed an increase in the score ( Figure 3 ).

**Figure 2:** – Risk of death according to the Rassi score on chest X-ray and echocardiography for definition of cardiomegaly in individuals with the cardiac form of Chagas disease; LVEDD: left ventricular end-diastolic diameter; CTR: cardiothoracic ratio.

**Figure 3:** – Individual risk of death according to the Rassi score on chest X-ray and echocardiography for definition of cardiomegaly in individuals with the cardiac form of Chagas disease; red dashed line and yellow dashed line correspond to a Rassi score of 12 (high risk) and 7 (moderate risk); LVEDD: left ventricular end-diastolic diameter; CTR: cardiothoracic ratio.

## Discussion

The present study demonstrated that there is a clear and significative relationship between detection of cardiomegaly by CTR and detection of left ventricular dilatation by echocardiography in a non-selected group of outpatients with diagnosis of CCC. These results open new perspectives to the use of LVEDD determined by resting transthoracic echocardiography in substitution for the estimation of CTR by chest X-ray, to determine the risk of death in patients with CCC using the Rassi score, whose results were eventually modified. This would prevent the use of radiological test, which requires radiation, despite low, and allow determining both left ventricular size and left ventricular systolic function by a single, non-invasive test that does not involve radiation. This perspective seems attractive, since the LVEDD determined by echocardiography was shown to be a highly specific parameter to distinguish between the presence and absence of cardiomegaly in patients with chronic CD.

Our findings corroborate previous observations reported by Pereira-Barreto et al.^8^in 1983, of chest X-ray and echocardiographic results of a smaller sample (n=22), showing a good correlation between CTR values and left ventricular function.^8^On the other hand, in a study published in 2003, Perez et al.,^9^conducted a comparative analysis between posteroanterior chest X-ray and resting echocardiographic results, and showed a poor correlation between the tests for LVEDD and LVEF results. Also, in this study,^9^CTR did not show high sensitivity or positive predictive value in detecting left ventricular dysfunction. Therefore, despite its high specificity in detecting left ventricular systolic dysfunction, a CTR > 0.5 would not be useful in assessing this condition in CD, due to its low sensitivity in detecting, on resting transthoracic echocardiography, left ventricular dilatation or left ventricular systolic dysfunction by altered LVEF.

The reasons of the discrepancies between these studies are not clear. In the study by Perez et al.^9^and in our study, although the population sample consisted of outpatients with CCC attending university hospital, there were differences between them. In our study, there were almost no exclusion criteria, except for those related to the availability of CTR and echocardiographic results within a one-year period. In contrast, the broad exclusion criteria used in consecutive patients in the study by Perez et al.^9^may have resulted in a highly selected sample that may not be representative of CD patients. Thus, in their study,^9^only 28% and 29% of patients had increased CTR and left ventricular dysfunction, respectively, and the mean LVEF was 61% (vs. 51% in our study). These aspects indicate that our population with CCC were more severely ill, especially considering that only 8% of them had the indeterminate form of CD. In addition, it is plausible that differences in the chest X-ray results regarding CTR values between the two studies are more apparent than real. In fact, in the study by Perez et al.,^9^patients were divided into two groups by CTR values (normal vs. abnormal, p<0.05), and results showed a significant association (p<0.05) of LVEF reduction with increase in ventricular diastolic dimension and left ventricular segmental dysfunction, as evidenced by the left ventricular wall motion index.

It is worth pointing out that the results of the present study indicate that the absence of cardiomegaly on chest X-ray does not discard cardiac involvement when patients are assessed by methods able to provide better anatomic and functional details, comparable with resting transthoracic echocardiography. Thus, despite specific, chest X-ray has low sensitivity in detecting cardiac involvement in CD patients, and its use as screening method or diagnostic criterion for the indeterminate form of CD may be questionable. In this context, although the indeterminate form of CD is still defined in current guidelines based on a normal CTR on chest X-ray,^3 , 10 , 11^substitution of this criterion with normal resting echocardiogram was already proposed in 2002.^12^

The Rassi score is universally regarded as the most valuable instrument for the establishment of the vital prognosis of patients with CCC. This is a robust score, developed by a multivariate analysis of many risk factors of death in CCC, externally validated in other independent cohorts. However, in the study by Perez et al.,^9^variables were analyzed in a dichotomous rather than continuous manner, which gives room for complementation. Among the variables analyzed in a non-continuous manner, there was the LVEDD, conventionally measured by echocardiography. However, when LVEDD was assessed in dichotomized categories, *i.e* ., presence or absence of increase, may be not appropriate for the evaluation of patients with CCC due to the typical segmental myocardial abnormality of the disease.^1^

Results of the present study are in line with the importance of echocardiography as an essential instrument for the follow-up of patients with chronic CD, especially of those with early myocardial deficits of CCC.^10^Echocardiography allows not only confirmation (or not) of cardiomegaly in case of questionable findings on chest X-ray, but also analysis of cardiomyopathy marked by regional changes of ventricular contractility that result from early and prominent disturbances in the natural course of the underlying disease.^13^Also, detection of these regional abnormalities at early stages of CCC has prognostic and therapeutic implications, in light of recent reports showing that even minimal changes in the left ventricular segmental wall motion index when left ventricular systolic function is preserved are determinant of severe outcomes including mortality.^13^

In the seminal study by Rassi Jr. et al.,^5^the cohort included in the multivariate analysis and development of the score consisted of 424 patients, most of them at low risk (61%), while 19% and 20% of the patients were at moderate and high risk, respectively. In the present study, we found a more balanced distribution of patients into the risk groups – 36%, 33% and 31% at low, intermediate, and high risk, respectively. Similarly, when LVEDD is substituted for CTR in the score, there was a small increase in the proportion of patients at low risk (40%) at the expense of a slight reduction in the percentage of patients at intermediate risk (28%). Therefore, further studies with clinical follow-up are warranted for validation of the Rassi score modified by substitution of the CTR as the radiological parameter of cardiac dilatation with the more specific echocardiographic parameter of left ventricular dilatation, and determination of the prognosis impact of such modification.

### Limitations

The present study has some limitations. The tests compared (chest X-ray and echocardiography) were not conducted on the same day, similar to the other tests included in the Rassi score, which were performed on different days. In any case, efforts were made to minimize confounding factors and factors associated with clinical changes, to assure that no change in clinical status or in drug therapy of patients occurred in the interval between the tests. Right atrial dimensions were not measured, thereby limiting the assessment of dilation of this chamber as a discordant factor between echocardiography and chest X-ray. Finally, although patients were not followed longitudinally for the prognostic assessment of LEVDD by echocardiography, they have been followed-up at an outpatient clinic and will be reassessed for this parameter in the future.

## Conclusions

LVEDD measured by echocardiography is an appropriate parameter to distinguish between the presence and absence of cardiomegaly on chest X-ray, with high specificity in patients with chronic CD. Substitution of echocardiographic LVEDD for the radiological CTR in the Rassi score was shown to be feasible and did not cause substantial change in the scores obtained. These results open new perspectives to avoid the use of a test that involves radiation and, rather, use a single test (echocardiography) to measure two components of the Rassi score – cardiac dimension and ventricular systolic function. The potential prognostic role of this modified Rassi score is a worthy subject for future studies.
